# Precarious employment and its impact on chronic obstructive pulmonary disease in the Swedish working population – a longitudinal register-based study

**DOI:** 10.5271/sjweh.4319

**Published:** 2026-11-01

**Authors:** Filippa Lundh, Virginia Gunn, Bertina Kreshpaj, Signild Kvart, Theo Bodin, Maria Albin, Jenny Selander, Sandra Ekström, Pernilla Wiebert, Nuria Matilla-Santander

**Affiliations:** 1 Institute of Environmental Medicine, Karolinska Institutet, Stockholm, Sweden.; 2 School of Nursing, Cape Breton University, Nova Scotia, Canada.; 3 Section of Epidemiology, Department of Public Health, University of Copenhagen, Copenhagen, Denmark.; 4 Center for Occupational and Environmental Medicine, Region Stockholm, Stockholm, Sweden.; 5 Department of Clinical Science and Education Södersjukhuset, Karolinska Institutet, Stockholm, Sweden.

**Keywords:** COPD, job exposure matrix, register-based research, Sweden

## Abstract

**Objective:**

Chronic obstructive pulmonary disease (COPD) is a respiratory disorder, and – after tobacco smoking – occupational exposures are among the most important risk factors. Precarious employment (PE) has been linked to a range of adverse mental and physical health outcomes; however, evidence regarding its potential association with COPD remains limited. PE may contribute to COPD risk through both greater exposure to occupational hazards and health-related behaviors, particularly smoking. This study aimed to examine whether PE is associated with COPD in the Swedish working population and assess whether these associations differ by gender.

**Methods:**

A nationwide register-based cohort of 1 984 081 working-age individuals in Sweden was followed for 14 years (2003–2017). PE was operationalized multidimensionally: contractual employment insecurity, temporariness, multiple jobholding, income level, and coverage by collective bargaining agreements. Cumulative incidence of COPD (2004–2017) was identified through register-based diagnoses. Risk ratios (RR) were estimated using generalized linear models with a log link function, adjusting for sociodemographic factors, occupational exposures, and smoking where available.

**Results:**

PE was associated with a graded increase in COPD risk, ranging from substandard employment [adjusted RR (RR_adj_) 1.18, 95% confidence interval (CI) 1.14–1.22] to PE (RR_adj_ 1.39, 95% CI 1.31–1.47). Among the dimensions of PE, low income was the strongest contributor (RR 1.44, 95% CI 1.39–1.50), while unstable employment had a modest effect (RR 1.16, 95% CI 1.12–1.20).

**Conclusion:**

PE is associated with elevated risk of COPD in the Swedish workforce. These findings emphasize the role of employment conditions as a social determinant of respiratory health.

Chronic obstructive pulmonary disease (COPD) is a chronic respiratory condition characterized by persistent airflow limitation, leading to breathing difficulties and a progressive decline in lung function (1). The disease results from an abnormal inflammatory response of the lungs to harmful airborne agents, such as noxious gases and particles (2). COPD is a major cause of morbidity, mortality, and healthcare use worldwide, including in Sweden, where the prevalence has been estimated at approximately 7% (3, 4). Globally, COPD is the fourth leading cause of death, and mortality is expected to continue to rise (4).

In high-income countries, the primary risk factors for COPD are tobacco smoking and exposure to airborne particles, including occupational exposures and ambient air pollution (1, 5). Tobacco smoking remains the dominant cause, with estimates suggesting that up to 50% of smokers develop COPD by the age of 75 (6, 7). However, smoking rates have declined in Sweden in recent decades, which is expected to reduce the burden of smoking-related COPD while increasing the relative contribution of other risk factors (3, 7). Indeed, approximately 30% of COPD cases occur among never-smokers, with exposure to dust, fumes, and gases identified as key contributors in this group (1, 3, 8).

In this context, occupational exposures to airborne particles become increasingly important determinants of COPD. However, exposure to occupational hazards is not solely determined by job tasks but is also shaped by broader employment conditions. Poor employment conditions are often conceptualized as precarious employment (PE), a multidimensional construct describing employment characterized by instability, inadequate income, and limited rights and protections (9, 10). Importantly, while structurally associated with traditional socioeconomic indicators like education, PE captures distinct, relational labor-market dynamics and proximal workplace stressors that represent an independent social determinant of health.

PE has become more prevalent in recent decades due to structural changes in labor markets, including increased flexibility and the decline of standard employment relationships (11, 12). As a growing and well-recognized social determinant of health, PE has been linked to a range of adverse outcomes, including mental illness, cardiovascular disease, mortality, musculoskeletal disorders, and occupational injuries (13–15).

From a conceptual standpoint, there are several mechanisms through which PE may increase the risk of COPD. Workers in PE may be more frequently exposed to occupational hazards such as dust and fumes, have limited access to protective equipment, and experience reduced regulatory protection in terms of occupational safety (6, 16, 17) In addition, PE may influence health-related behaviors. For example, studies suggest that PE increases smoking through pathways related to economic insecurity, stress, and reduced job control, while also embedding smoking in workplace cultures as a coping strategy and a means of accessing breaks and social interaction (18, 19). Importantly, these pathways are highly gendered due to horizontal labor market segregation and varying smoking behaviors by sex in Sweden (20). This suggests that the health impacts of precarious employment may manifest differently for men and women.

Despite these plausible pathways, empirical evidence directly linking PE to COPD remains scarce (21). This represents a critical gap in understanding both the impact of changing labor market conditions on health and the determinants of COPD. While evaluating these specific mediating mechanisms falls outside the scope of the current work, establishing whether an overarching relationship exists is a necessary first step. Therefore, given the increasing prevalence of PE and its relevance as a social determinant of health and health inequalities, the aim of this study is to investigate whether PE is associated with the risk of developing COPD in the Swedish working population, and whether this association differs by sex.

## Method

### Study design and data collection

This register-based cohort study followed participants from baseline in 2003 through 2017. The exposure variable was the level of PE, determined at baseline for the year 2003.

The health outcome is the cumulative incidence of COPD diagnosis during the follow-up period (2004-2017). Data for the cohort were obtained from four population-based registers: (i) longitudinal integrated database for health insurance and labor market studies (LISA), (ii) the national patient-register, (iii) the Swedish military conscript register (SMCR), and (iv) the Swedish Medical Birth Register (SMBR) (22–24).

The study population was derived from individuals engaged in the labor market at baseline and meeting the following inclusion criteria: (i) aged 40–65 years in 2003, and (ii) having complete data on the exposure variable. The selected age range reflects the working-age population and ensures sufficient risk of developing COPD during the follow-up period, given the long latency of the disease and its typical onset in mid-life.

Participants were excluded if they: (i) had a recorded diagnosis of COPD in inpatient or outpatient registers prior to baseline (to reduce the risk of reverse causation); (ii) experienced prolonged unemployment (>6 months) during 2003 (to distinguish the effects of precarious employment from those of unemployment and to maintain a study population attached to the labor market); (iii) had income levels of <100 SEK in 2003; (iv) received student or pension benefits, as these groups differ from the target working population and may introduce exposure misclassification; and (v) were self-employed in 2003, due to the heterogeneity of this group and the challenges in consistently classifying their employment conditions within the exposure framework.

The final analytical sample comprised 1 984 081 individuals (figure 1).

**Figure 1:**
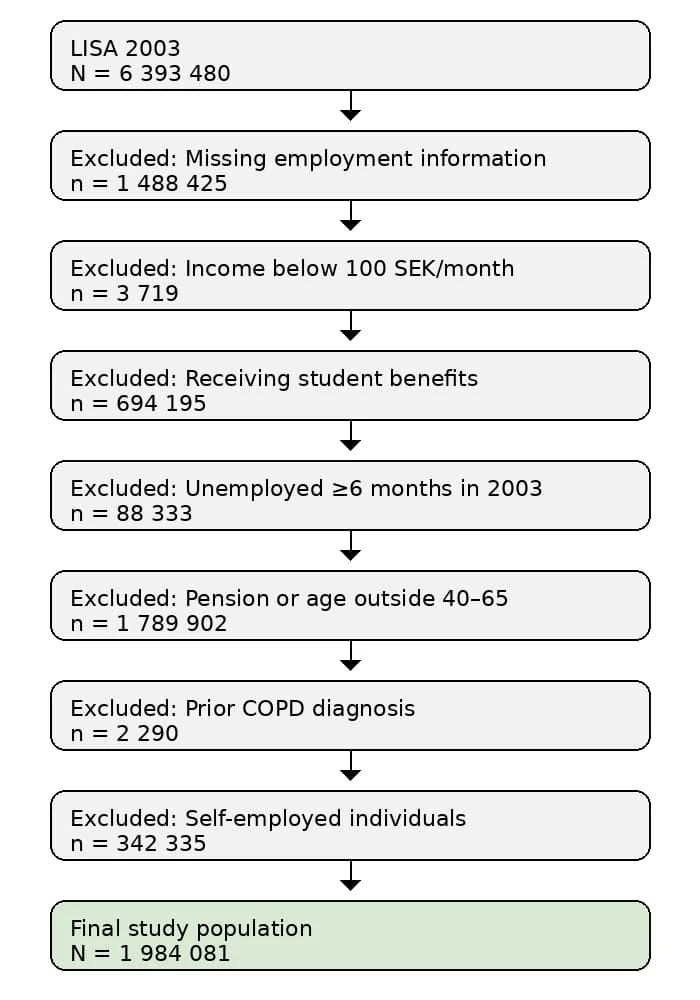
Flowchart of the study population selection and analytical sample.

### Study variables

*Exposure variable*. The exposure variable used was level of employment precariousness, measured with the Swedish Register Operationalization of Precarious Employment (SWE-ROPE) (10) using the LISA register data. The exposure variable was the level of PE, determined at baseline for the year 2003. SWE-ROPE covers three dimensions of precariousness: employment insecurity, income inadequacy, and lack of rights and protection as identified by Kreshpaj et al (9). For each of these dimensions, five items of PE are operationalized: contractual employment insecurity, temporariness, multiple jobholding, income level, and coverage under collective bargaining agreements (see supplementary material, XXX, table S1). Every individual was given a score for each item. The sum of all these items results in a PE score of -3–9, with a lower score indiciating worse employment conditions.

Based on the PE score, the exposure variable was created and grouped into categories: (i) standard employment relationship (SER) (score ≥0 points); (ii) substandard employment relationship (subSER): (score -1– -3 points); PE (score < -3 points).

PE was treated as categorical variables, in line with theoretical frameworks that conceptualize precariousness as distinct profiles of labor market disadvantage rather than a continuous score. This categorization also facilitates the translation of findings into actionable occupational health policies.

*Outcome variable.* The outcome variable is the cumulative incidence of COPD between 2004-2017. COPD cases are classified according to the following ICD-10 codes from registers: J44.0 (COPD with acute lower respiratory tract infection), J44.1 (COPD with acute exacerbation, unspecified), J44.8 (other specified COPD), and J44.9 (COPD, unspecified) (ICD-10, version, 2019).

*Confounding variables.* Potential confounders were identified a priori using a directed acyclic graph (DAG) (supplementary figure S2) (25). Based on the assumed causal structure (supplementary figure S2), a minimal sufficient adjustment set was defined. This included sociodemographic factors obtained through the LISA register such as age at baseline (40–48, 49–56, and 57–65 years), educational attainment (elementary/high school, higher education <3 years, and ≥3 years), and country of origin (Swedish- versus non-Swedish-born). Socioeconomic position was further captured by family income, categorized into quintiles (<60%, 60–79%, 80–119%, 120–200%, and >200% of the reference level) (26). Days in unemployment in 2003: 0–30, 30–90, and 90–180 days) were also adjusted for as shorter unemployment spells may form part of trajectories of precariousness and could confound the association between PE and COPD. Based on our causal framework (DAG), traditional socioeconomic markers like educational attainment were treated strictly as baseline confounders rather than mediators or colliders, as education temporally precedes labor market entry and acts as a common cause of both PE selection and long-term respiratory health.

*Confounder-mediator overlap*. Smoking and occupational exposure to chemicals and airborne particles were considered variables with potential confounder–mediator overlap in the association between PE and COPD, however, mediation analysis was not possible due to smoking data were collected prior to baseline, precluding temporal ordering with the exposure, and occupational exposure to chemical and airborne particles exposure was only available at an aggregated level, limiting individual-level inference.

Smoking is a well-established risk factor for COPD and may be associated with PE through prior socioeconomic conditions, thus acting as a confounder. At the same time, smoking may also lie on the causal pathway between PE and COPD, as PE may influence smoking behavior through mechanisms such as economic insecurity, stress, and reduced job control, thus acting as a mediator. Therefore, smoking was treated as a variable that may act both as a confounder and a mediator, considering the limitations of the available data.

Smoking status, available only for a subpopulation, was included as a binary variable (smoker versus non-smoker). Data came from two sources: the SMCR for men (22) and the SMBR for women (23) These sources differed in timing and context of measurement. The SMCR recorded tobacco use at military conscription in 1969 (approximately 18 years old), whereas the SMBR recorded maternal smoking at the first antenatal visit between 1983 and 2003. Because smoking was measured differently among men and women, analyses were stratified by sex. Analyses including smoking were limited to those with available data (N=33 277 men; N=391 744 women). Owing to differences in measurement context and restriction to a subpopulation, the smoking variable may be subject to misclassification.

Similarly, occupational exposure to chemicals and airborne particles was considered a variable with potential confounder–mediator overlap. Such exposures are directly associated with COPD risk and may also be patterned by prior socioeconomic position, thereby acting as confounders. At the same time, occupational exposures may lie on the causal pathway between PE and COPD, as PE may influence the likelihood of being employed in hazardous occupations and access to workplace protections, thus acting as mediators.

Occupational exposure to airborne substances in 2000–2003 was assessed using SweJEM, a Swedish quantitative job exposure matrix (JEM) (27). SweJEM provides information on exposure by occupation coded according to the Swedish version of the International Standard Classification of Occupations (ISCO) from the 1950s to today. Seven substances identified as occupational risk factors for COPD were included (supplementary table S2). Exposure was operationalized as a binary variable (exposed versus unexposed), using a threshold of ≥50% probability of exposure within a given occupation.

### Statistical analysis

Descriptive statistics were calculated to examine the distribution of PE and its components in relation to cumulative COPD incidence. Covariates were included to characterize the study population. Analyses were performed for the total population and stratified by sex.

For the main analysis, generalized linear models with a binomial family and log link were used to estimate crude and adjusted risk ratios (RR) for cumulative incidence of COPD. A generalized linear model (GLM) was used instead of a time-to-event approach because baseline precarious employment was conceptualized as a proxy for an accumulated lifetime trajectory rather than an acute exposure. Furthermore, given the long latency period and gradual onset of COPD, our objective was to assess cumulative 14-year incidence rather than the exact timing of clinical diagnosis. Model specification was guided by a DAG. Because the precise temporal ordering of smoking and occupational exposures relative to baseline PE cannot be definitively established with the available register data, these variables present potential confounder–mediator overlap. We therefore estimated a series of models to assess the total effect of PE under differing causal assumptions.

Model 1 adjusted solely for sociodemographic factors (age, sex, educational level, family income, number of days in unemployment, and migration status). This model conceptualizes smoking and occupational exposures as downstream mediators; under this causal assumption, these variables were omitted from the model to capture the unattenuated total effect of PE.

Model 2 additionally adjusted for occupational exposure to chemicals and particles using a JEM, treating these exposures instead as potential baseline confounders to estimate the adjusted total effect under this alternative scenario.

Model 3 further adjusted for smoking status in the subpopulation with available data, thereby treating both variables as baseline confounders. The smoking variable was measured differently for women and men, and this was accounted for in the analysis. Due to the low number of COPD cases in this subpopulation and resulting model instability, Poisson regression with robust variance estimation was used instead of binomial regression to estimate RR.

Sensitivity analyses were conducted to assess the robustness of the findings. First, each dimension of PE was examined separately as the exposure, with adjustment for sociodemographic factors. Second, analyses were stratified by age groups to explore potential effect modification. Lastly, analyses were performed in the population unexposed to chemicals and fumes.

All analyses were performed using Stata version 17.

## Results

### Socio-demographic characteristics, exposure to particles and smoking by levels of precariousness

Table 1 presents the sociodemographic characteristics of the study population. The study population consisted of 1 984 081 individuals, including 977 461 men and 1 006 620 women. Overall, 62% were in SER, 33% in subSER, and 5% in PE.

**Table 1:** Levels of exposure to precarious employment for men and women by socio-demographic characteristics at baseline (2003), incidence of chronic obstructive pulmonary disease (COPD) (2004–2017) and exposure to particles/chemicals in the workplace and smoking. [SER=standard employment relationship; PER=precarious employment relationship; JEM=job exposure matrices.]

	Total (N)	Men (N=977 461)					Women (N=1 006 620)			
		N	SER (%)	Substandard (%)	PER (%)		N	SER (%)	Substandard (%)	PER (%)
Total population	1 984 081		603 747 (62)	320 410 (33)	53 304 (5)			423 420 (42)	523 000 (52)	60 200 (6)
COPD cases	17 832									
Cases (cumulative incidence 2004–2017)			3464 (0.57)	3224 (1.00)	773 (1.45)			3272 (0.77)	6174 (1.18)	925 (1.53
Age (years)										
40–48			230 562 (38)	125 385 (39)	18 956 (35)			150 287 (35)	195 578 (38)	23 571 (39)
49–56			125 385 (39)	102 726 (32)	14 731 (28)			162 130 (38)	170 938 (33)	17 359 (29)
57–65			18 903 (35)	92 299 (29)	19 617 (37)			111 003 (26)	156 484 (30)	19 270 (32)
Level of education										
Elementary/ high school			387 896 (64)	242 769 (76)	40 938 (78)			204 759 (48)	402 349 (77)	46 612 (78)
Higher education <3 years			88 651 (15)	35 842 (11)	5 389 (10)			91 036 (22)	63 374 (12)	6697 (11)
Higher education >3 years			126 057 (21)	40 999 (13)	6 733 (13)			127 321 (30)	56 635 (11)	6754 (11)
Missing		2187					1083			
Country of origin										
Swedish born			548 913 (91)	281 155 (88)	44 195 (83)			381 859 (90)	463 301 (89)	51 421 (85)
Non-Swedish born			54 829 (9)	39 252 (12)	9108 (17)			41 560 (10)	59 691 (11)	8778 (15)
Missing		9					10			
Family income (%)										
<60			91 627 (15)	88 827 (28)	22 901 (43)			83 614 (20)	137 064 (26)	22 127 (37)
60–79			71 036 (12)	48 321 (15)	9 237 (17)			53 353 (12)	81 872 (16)	10 504 (17)
80–119			236 412 (39)	119 831 (37)	15 274 (29)			138 020 (33)	201 508 (39)	18 772 (31)
120–200			178 179 (30)	57 843 (18)	5122 (10)			129 817 (31)	92 548 (17)	7602 (13)
>200			26 480 (4)	5549 (2)	719 (1)			18 608 (4)	9979 (2)	1175 (2)
Missing		103					57			
Days in unemployment										
0–30			596 616 (99)	300 071 (94)	44 770 (84)			420 734 (99)	507 016 (97)	52 140 (87)
30–90			3521 (>1)	9593 (3)	3963 (7)			1477 (>1)	8377 (2)	4048 (7)
90–180			3610 (>1)	10 746 (3)	4571 (8)			1209 (>1)	7607 (1)	4012 (7)
Occupational exposure to particles (JEM)										
Exposed			81 263 (13)	57 675 (18)	9 177 (17)			5716 (1)	9425 (2)	1438 (2)
Unexposed			522 484 (87)	262 735 (82)	44 127 (83)			417 704 (99)	58 762 (98)	58 762 (98)
Smoking (N=425 021)										
Men with smoking data		33 277								
Nonsmoker			9892 (45)	4072 (41)	506 (39)					
Smoker			12 129 (55)	5900 (59)	778 (61)					
Women with smoking data							391 744			
Nonsmoker								131 826 (79)	151 442 (75)	16 277 (72)
Smoker								34 671 (21)	51 090 (25)	6438 (28)

With respect to covariates, similar patterns were observed among men and women, with individuals in PE more frequently having lower levels of education, lower family disposable income, a higher proportion of non-Swedish origin, and more days in unemployment compared to those in SER.

Occupational exposure to particles was more frequent among men in PE (17%) compared to SER (13%), while among women, exposure was low overall but slightly higher in PE (2%) compared to SER (1%). Among individuals with available smoking data, smoking prevalence was higher in PE. Among men, 61% of those in PE were smokers, compared to 59% in subSER and 55% in SER. Among women, smoking prevalence was 28% in PE, compared to 25% and 21%, respectively.

### Cumulative incidence of COPD between 2004–2017

Increasing levels of PE (from SER to subSER to PE) were accompanied by higher cumulative incidence of COPD (2004–2017) among both men and women, with slightly higher incidence observed among women in PE. Among men, the incidence was 0.57% in SER, 1.00% in subSER, and 1.45% in PE. Among women, the corresponding values were 0.77%, 1.18%, and 1.53%, respectively (table 1).

### Associations between levels of precariousness and incidence of COPD

In the total population, both subSER and PE were associated with higher RR for COPD compared with SER (table 2). Adjustment for sociodemographic factors (model 1) attenuated the estimates compared with crude analyses, and further adjustment for occupational exposure to particles and chemicals (model 2) resulted in minimal additional attenuation. For example, in the total population, the RR for PE decreased from 2.28 (95% CI 2.16–2.40) in crude analyses to 1.42 (95% CI 1.34–1.50) in model 1 and 1.39 (95% CI 1.31–1.47) in model 2. A similar pattern was observed for subSER.

**Table 2a:** Associations between exposure to levels of precarious employment (PE) (2003) and risk of chronic obstructive pulmonary disease (COPD) (2004–2017) **for the total population**. [RR=risk ratio; CI=confidence level.]

Levels of PE	Total population N=984 081				
	Crude (N=1 984 081)		Model 1 ^a^ (N=1 980 638)		Model 2 ^b^ (N=1 980 638)
	RR (95% CI)		RR (95% CI)		RR (95% CI)
SER ^c^	Ref		Ref		Ref
SubSER ^d^	1.69 (1.64–1.75)		1.20 (1.16–1.24)		1.18 (1.14–1.22)
PE ^e^	2.28 (2.16–2.40		1.42 (1.34–1.50)		1.39 (1.31–1.47)

**Table 2b:** Associations between exposure to levels of PE (2003) and risk of chronic obstructive pulmonary disease (COPD) (2004–2017) **for men**.

Levels of PE	Crude (N=977 461)		Model 1 ^a^ (N=975 164)		Model 2 ^b^ (N=975 164)
	RR (95% CI)		RR (95% CI)		RR (95% CI)
SER ^c^	Ref		Ref		Ref
SubSER ^d^	1.75 (1.67–1.83)		1.33 (1.26–1.39)		1.30 (1.24–1.37)
PE ^e^	2.52 (2.33–2.73)		1.53 (1.41–1.66)		1.49 (1.38–1.62)

**Table 2c:** Associations between exposure to levels of PE (2003) and risk of chronic obstructive pulmonary disease (COPD) (2004–2017) **for women**.

Levels of PE	Crude (N=1 006 620)		Model 1 ^a^ (N=1 005 474)		Model 2 ^b^ (N=1 005 474)
	RR (95% CI)		RR (95% CI)		RR (95% CI)
SER ^c^	Ref		Ref		Ref
SubSER ^d^	1.52 (1.46–1.59)		1.09 (1.04–1.14)		1.08 (1.03–1.12)
PE ^e^	1.98 (1.84–2.13)		1.28 (1.19–1.38)		1.25 (1.16–1.35)

^a^ Adjusted by age, sex, level of education, days in unemployment during the last year, family income as percentage of the median income. ^b^ Adjusted by age, sex, level of education, days in unemployment during the last year, family income as percentage of the median income and exposure to chemicals and fumes (JEM). ^c^ Standard employment relationship score: 0 or <0. ^d^ Substandard employment relationship score: -1– -3. ^d^ Precarious employment score: < -3.

Sex-stratified analyses showed comparable patterns. Among both men and women, adjustment for sociodemographic factors (model 1) reduced the magnitude of the associations, with minimal additional attenuation after accounting for occupational exposures in model 2. In model 2, associations remained elevated among both sexes, with higher RR observed among men than women. For PE, the RR was 1.49 (95% CI 1.38–1.62) among men and 1.25 (95% CI 1.16–1.35) among women.

In analyses restricted to individuals with available smoking data (model 3), further attenuation of the estimates was observed after adjustment for smoking (table 3). Among men (N=33 277), the adjusted RR (RR_adj_) was 1.21 (95% CI 0.91–1.61) for subSER and 1.63 (95% CI 0.97–2.71) for PE (table 3). Among women (N=391 744), the corresponding estimates were 1.34 (95% CI 1.20–1.49) for subSER and 1.71 (95% CI 1.43–2.04) for PE (table 3). Due to the smaller number of COPD cases in this subpopulation, particularly among men, these estimates should be interpreted with caution.

**Table 3:** Associations between exposure to levels of precarious employment (PE) (2003) and risk of chronic obstructive pulmonary disease (COPD) (2004–2017) among individuals with complete data on smoking ^a^, separated by sex.

	Men					Women			
	Total (N)	Cases N (%)	Crude (N=33 277)	Model 3 ^b^ (N=33 247)		Total (N)	Cases N (%)	Crude (N= 391 744)	Model 3 ^b^ (N= 391 490)
SER ^c^	21 907	114 (0.52)	Ref	Ref		166 497	498 (0.30)	Ref	Ref
SubSER ^d^	9972	85 (0.85)	1.64 (1.24–2.17)	1.21 (0.91–1.61)		202 532	1075 (0.53)	1.77 (1.59–1.97)	1.34 (1.20–1.49)
PE ^e^	1284	18 (1.40)	2.70 (1.65–4.43)	1.63 (0.97–2.71)		10 715	182 (0.80)	2.68 (2.26–3.17)	1.71 (1.43–2.04)

^a^ Complete smoking data is available for men from the Swedish Conscription register for the year of (1969), and the smoking data for women is available from the Swedish Medical Birt register (SMBR) and for the years 1983–2003. ^b^ Adjusted by age, sex, level of education, days in unemployment during the last year, family income as percentage of the median income and exposure to chemicals and fumes (JEMS) and smoking habits. ^c^ Standard employment relationship score: 0 or <0. ^d^ Substandard employment relationship score: -1– -3. ^d^ Precarious employment score: < -3.

### Sensitivity analyses

When examining the individual dimensions of PE, lower income levels and unstable employment were associated with higher risk of COPD (supplementary table S2), whereas no clear associations were observed for coverage by collective bargaining agreements or temporary agency employment. Holding jobs across multiple sectors was associated with a lower risk of COPD.

When stratifying by age group, higher RR were observed in all age groups, with stronger associations among younger individuals (40–48 years) and progressively lower estimates in older age groups (supplementary table S4). For PE, the RR_adj_ (model 2) was 1.79 (95% CI 1.54–2.09) in the youngest group, 1.58 (95% CI 1.42–1.75) among those aged 49–56 years, and 1.31 (95% CI 1.24–1.42) among those aged 57–65 years. A similar pattern was observed for subSER.

Results were similar after restricting the analysis to individuals without occupational exposure to particles and chemicals (supplementary table S6). For PE the RR_adj_ (model 1) was 1.50 (95% CI 1.37–1.64) for men and 1.26 (95% CI 1.17–1.36) for woman. For subSER, the RR (model 1) was 1.34 (95% CI 1.26–1.41) for men and 1.08 (95% CI 1.03–1.13) for women.

## Discussion

### Main findings

PE was associated with a higher risk of COPD compared with standard employment relationships. The risk increased across levels of precariousness (from SER, to subSER, to PE) for both men and women, with consistently higher estimates among men across models. Adjustment for sociodemographic factors reduced the magnitude of the associations, while additional adjustment for occupational exposure to particles and chemicals resulted in minimal further attenuation.

In analyses restricted to individuals with available smoking data, further attenuation was observed after adjustment for smoking, although elevated risk persisted, particularly among women. When stratified by age, associations were present across all age groups, with higher estimates among younger individuals and progressively lower estimates with increasing age. When restricted to individuals without occupational exposure to chemicals and fumes, the pattern remained with a stronger association for men. Analyses of the individual dimensions of PE indicated that lower income and unstable employment were the dimensions most consistently associated with higher risk.

### Interpretation of the results

Our findings are consistent with previous research. Earlier studies examining the relationship between work arrangements and COPD mainly focused on occupational exposures, but with one review linking PE to respiratory disease and other adverse health outcomes (28–30). Existing research suggests that the association between PE and COPD may operate through both shared social determinants and more specific causal pathways. One study reported a higher prevalence of PE among individuals with more severe COPD, defining PE as temporary or daily employment and non-precarious work as standard employment, self-employment, or unpaid family work proposing that PE and COPD may partly reflect common socioeconomic determinants, including smoking, and indoor and outdoor air pollution (31). Another study conceptualized the relationship within a life-course framework, whereby low socioeconomic position is associated with established COPD risk factors, such as adverse early-life conditions, smoking, and environmental exposures, while simultaneously increasing the likelihood of entering PE (32). PE may in turn further contribute to COPD risk through cumulative disadvantage across the life course.

In this study, PE was measured at a single time point in 2003 among individuals aged 40–65 years, and incidence of COPD was assessed during follow-up from 2004 to 2017. As such, the exposure measurement reflects baseline employment conditions rather than cumulative or time-varying exposure to PE. Given the long latency period of COPD, the observed associations are therefore more likely to capture longer-term socioeconomic and employment trajectories, for which baseline PE may serve as a proxy, rather than the effects of short-term exposure alone.

Building on our findings and previous research, several pathways may theoretically explain the association between PE and COPD. First, precarious workers may experience higher exposure to occupational dusts, fumes, and chemicals, potentially combined with more limited access to protective equipment, safety training, and workplace prevention measures. However, the attenuation observed after adjustment for sociodemographic factors (model 1), together with the minimal additional attenuation after accounting for occupational exposures (model 2), suggests that the total association between PE and COPD remains robust and is largely independent of the occupational exposures to dusts and fumes captured via JEM in this study.

Due to limitations in the available data, formal mediation analyses could not be performed with smoking and exposure to fumes and chemicals. Smoking information was collected prior to baseline and is only available for a subsample, while occupational exposures were assessed at the occupational rather than individual level before baseline. Instead, sequential adjustment models were used to explore the sensitivity of our baseline estimates under different causal assumptions regarding whether these factors acted as mediators or confounders. The small attenuation after adjustment for occupational exposures, together with similar estimates in analyses restricted to individuals without such exposures, suggests that the observed PE–COPD association persists outside of accounted occupational particles and fumes.

Similarly, PE is knowingly associated with higher smoking prevalence as lower socioeconomic position, financial strain, and chronic stress may increase the likelihood of smoking as a coping strategy (18, 19). This notion is supported by the further attenuation seen after smoking adjustment in model 3. Although the RR were slightly attenuated compared with the main results, the dose–response pattern remained evident. These findings suggest that while we cannot empirically disentangle the precise mediating role of smoking, the persistence of an elevated risk supports the framework that PE is an independent risk factor for increased risk of COPD.

This suggest that PE captures a constellation of social and material conditions that may influence COPD risk over time, including differences in health behaviors and healthcare access, rather than acting solely through direct occupational exposures. Additionally, to smoking and occupational exposures, precarious workers may face barriers to healthcare access, including fewer opportunities to attend medical appointments, lower continuity of care, and delayed use of specialist services. COPD cases were identified from secondary and specialist care diagnoses. Early-stage COPD may be managed in primary care or remain undiagnosed, so milder cases may be missed, and the timing of clinical diagnosis can vary based on occupational demands (33).

This could contribute both to delayed diagnosis and more advanced disease at the time COPD enters specialist registers. Among the individual dimensions of PE, low income showed the strongest association with COPD risk, consistent with evidence linking economic hardship to poor health, and supports material deprivation as a central component of PE (34, 35). No statistically significant associations between COPD risk and low collective bargaining agreement coverage, or temporary or agency employment were observed. This finding suggests that these dimensions may be less relevant for COPD risk than economic deprivation. The absence of a protective effect of collective bargaining coverage contrasts with previous findings for other health outcomes, such as sickness absence (36) and occupational injuries (37). One possible explanation is that safety measures related to collective coverage may be insufficient to prevent COPD, or that non-covered workplaces implement comparable protective practices.

Finally, having multiple jobs was associated with a lower risk of COPD. This may reflect reduced cumulative exposure when work is distributed across sectors rather than concentrated in a single high-exposure occupation. Occupational exposures are estimated to account for approximately 19% of COPD cases overall and up to 30% among never smokers, with elevated risks observed in occupations such as construction, transportation, machine operation, and material handling (1, 3, 8). However, other explanations could be plausible and the risk of bias (multiple jobholders across sectors may be a positively selected group (younger and/or healthier) as well as those with early symptoms may be unable to hold multiple jobs.

The sex-stratified findings warrant cautious interpretation. Women showed higher cumulative COPD incidence than men within each PE category, yet the adjusted RR were consistently lower. Several factors may explain this. First, the female reference group (females in SER) had a higher baseline incidence than the male reference group, which automatically reduces RR contrasts even when absolute differences are similar. This is supported by the adjusted analyses (model 3) restricted to individuals with information of smoking data, where male estimates were more decreased after full adjustment, whereas female RR remained higher. Taken together, these considerations suggest that the apparently stronger associations among men should be interpreted cautiously, as sex differences in relative risks may partly reflect residual confounding, exposure misclassification, and differential ascertainment rather than true differences in risk. The same caution should be used when interpreting the results from the age stratified analyses. The lower RR observed in older age groups may reflect either a lower incidence due to baseline selection or differences in care pathways, whereby COPD diagnoses are more likely to be captured in specialized care at younger ages, while older individuals are more often managed in primary care.

### Strengths and limitations of the study

Key strengths of this retrospective longitudinal register-based study include the large nationwide cohort, extensive register coverage, long follow-up, and adjustment for multiple confounders: age, sex, education, family income, country of birth, occupational chemical exposure, and smoking. Occupational exposure was assessed using a JEM based on measured workplace exposures, providing substance-specific occupational probabilities, though some individual misclassification is possible. A further strength is the multidimensional operationalization of PE using five indicators across three dimensions, reducing misclassification versus single-item measures. The long follow-up was appropriate given COPD’s long latency (1).

The main limitation consisted in the quality of the smoking data: only available for a subsample, measured differently among women and men, and collected at time points that may not reflect baseline smoking. This is particularly important due to smoking being the strongest established COPD risk factor. For women, smoking data came from birth registers and reflected self-reported smoking in early pregnancy, excluding never-pregnant women, potentially not representing later adult habits, and likely underreported due to stigma. For men, smoking data came from military conscription records in 1969, around three decades before baseline, with one measurement in late adolescence/early adulthood, unlikely to capture cessation, relapse, or intensity changes. Given declining smoking prevalence in Sweden since the 1970s, male smoking data likely overestimated baseline smoking. Differences in source, timing, and context between sexes may have caused residual confounding and reduced comparability.

COPD cases were identified from secondary and specialist care diagnoses. Early-stage COPD may be managed in primary care or remain undiagnosed, so milder cases may be missed. Diagnostic accuracy also depends on spirometry access and clinical assessment, which may vary, though validation studies show acceptable accuracy in the Swedish Patient Register, with <10% misclassification (38).

Finally, population-based registers minimized recall and selection bias and enabled linkage of occupational, socioeconomic, and health data. However, smoking data in the subpopulation were self-reported and may be affected by recall and social desirability bias, causing underreporting or misclassification. Ethical risks were minimal because data were anonymized and securely stored, and the potential public health benefits outweighed harms.

### Concluding remarks

PE is associated with an increased risk of COPD. These associations persist after accounting for smoking, occupational exposures, and sociodemographic factors. Lower income also independently increases COPD risk. While our study design evaluated the total effect rather than specific causal pathways, the observed associations are likely supported by theoretical mechanisms including greater exposure to harmful substances in precarious jobs, limited access to protective equipment and safety training, and adverse lifestyle factors such as higher smoking prevalence. These findings underscore the substantial health risks of PE and highlight the need for further research employing formal mediation designs to explicitly clarify these underlying mechanisms and form policies to protect vulnerable workers.

## Supplementary material

Supplementary material 1 Supplementary material
