# Supplementary material for: Precarious employment and its impact on chronic obstructive pulmonary disease in the Swedish working population – a longitudinal register-based study
Source: Scand J Work Environ Health. 2026 Oct 26;52(6):714–24. doi: 10.5271/sjweh.4319 (PMC13738666; doi:10.5271/sjweh.4319)
Supplement: Supplementary file 1 [file SJWEH-52-6-714-s001.pdf]

Precarious employment and its impact on chronic obstructive pulmonary disease in the Swedish working population – a longitudinal register-based study[1]

by Filippa Lundh, MPH,[2] Virginia Gunn, PhD, Bertina Kreshpaj, PhD, Signild Kvart, PhD, Theo Bodin, MD, PhD, Maria Albin, MD, PhD, Jenny Selander, PhD, Sandra Ekström, PhD, Pernilla Wiebert, PhD, Nuria Matilla-Santander, PhD

1. Supplementary material
2. Correspondence to: Filippa Lundh, Unit of Occupational Medicine, Institute of Environmental Medicine, Karolinska Institutet, Solnavägen 4, 113 65 Stockholm, Sweden. [E-mail: Filippa.lundh@ki.se].

Table S1. Scores in the Swedish Register Operationalization of Precarious Employment (SWEROPE).

Items of precarious employment	Categories in each item and scores	Score range
Contractual relationship insecurity	- Directly employed (0p) - Temporary agency employed (-1p)	Min -1p Max 0p
Contractual temporariness	- Stable employment (0p) - Unstable employment (-2p) *Measured as a change in employer in the past 2 years or the current year*	Min -2p Max 0p
Multiple jobholding	- 1–2 jobs (0p) - ≥3 jobs (-1p) - ≥3 jobs in ≥3 sectors (-2p) *Measured based on number of jobs and sectors in the past 2 years or current year*	Min -2p Max 0p
Probability of coverage by collective bargaining agreement (CBA)	- >90% (0p) - 70–90% (-1p) - <70% (-2p) *Estimated based on sector, company size, ownership (public/private), and sex*	Min -2p Max 0p
Income (% of median income)	- >200% (2p) - 120–200% (1p) - 80–119% (0p) - 60–79% (-1p) - <60% (-2p) *Includes work income, social insurance, and unemployment benefits*	Min -2p Max 2p

Table S2. The adjusted and unadjusted risk ratio for developing COPD related to level of precariousness shown for the whole population and stratified by the five dimensions of PE.

	Crude RR	95% CI	Adjusted RR	95% CI
Percentage of median income				
>60%	2.255	2.179 - 2.334	1.443	1.387 - 1.501
60-79%	1.476	1.423 - 1.530	1.201	1.155 - 1.249
80-119%	Ref		Ref	
120-200%	0.557	0.528 - 0.587	0.854	0.806 - 0.905
>200%	0.306	0.268 - 0.350	0.735	0.636 - 0.580
Contractual temporariness				
Stable	Ref		Ref	
Unstable	1.076	1.039 - 1.115	1.159	1.116 - 1.204
Probability to be covered by a collective bargaining agreement				
<70%	1.236	1.128 - 1.354	1.025	0.930 - 1.130
70-90%	Ref		Ref	
>90%	0.957	0.909 - 1.008	1.040	0.985 - 1.099
Contractual insecurity				
Directly employed	Ref		Ref	
Temporary agency employed	1.466	0.924 - 2.326	1.053	0.792 - 1.399
Multiple jobholding				
1-2 Jobs	Ref		Ref	
3 or more jobs	0.726	0.668 - 0.790	0.965	0.883 - 1.055
3 or more jobs in 3 or more sectors	0.687	0.610 - 0.774	0.843	0.747 - 0.952

Note: RR Adjusted by age, sex, level of education, days in unemployment during the last year, family income as percentage of the median income and exposure to chemicals and fumes (JEM) and the other items of PE).

Table S3. Chemical exposures associated with COPD from the SWEJEM.

Agent	Definition
DIESEL ENGINE EXHAUST	Occupational inhalation exposure to diesel engine exhaust, including exposure to: nitrogen dioxide (NO2), other nitrous oxides (NOX), carbon monoxide (CO), volatile and semi volatile hydrocarbons, inorganic and organic lead compounds, particulate PAHs (PAHP), volatile PAHs (PAHV), nitrated PAHs (nitroarenes), total particulate matter (TPM), aldehydes (ALDH).
OIL MIST	Occupational, inhalation exposure to untreated, treated or synthetic metal processing or drilling oils or fluids
PLANT DUST	Occupational, inhalation exposure to dust from plants (raw cotton, corn, flax, unground grain, malt, jute, leaves of trees etc), fruits (including fruit trees), vegetables (including herbs and spices), or plant borne materials (natural rubber, coffee, cocoa, peat, soya, sugar). Does not include wood dust (see WOOD), flour dust (see FLOU) or textile dust (eg processed cotton, see TEXT)
PULP OR PAPER DUST	Occupational, inhalation exposure to pulp or paper dust (pulp, newsprint, printing paper, soft papers, cardboard etc)
QUARTZ DUST	Occupational, inhalation exposure to respirable (aerodynamic diameter <5um) quartz or crystalline silica containing dusts (eg, granite). Does not include amorphous silica dust.
WELDING FUME	occupational exposure to welding fumes
WOOD DUST	Occupational, inhalation exposure to wood dust (pine, spruce, birch, other softwoods and hardwoods, cane, wood bark etc)

Table S4. Associations between exposure to levels of PE (2003) and risk of COPD (2004-2017) for the total population, stratified by age groups (40 – 48, 49 – 56, 57 – 65).

		Total population<br>N = 1. 984 081	
	**Crude**	**Adjusted model 1 (n)**	**Adjusted model 2 (n)**
		**Age group 40-48**	
	**Crude**<br>n = 744 339	**Adjusted model 1**<br>n = 742 712	**Adjusted model 2**<br>n = 742 712
	RR [95% CI]	RR [95% CI]	RR [95% CI]
SER	Ref	Ref	Ref
Substandard	1.93 [1.76 – 2.12]	1.35 [1.23 – 1.49]	1.36 [1.23 – 1.49]
PE	2.98 [2.57 – 3.44]	1.79 [1.53 – 2.09]	1.79 [1.54 – 2.09]
		**Age group 49 - 56**	
	**Crude**<br>n = 682 559	**Adjusted model 1**<br>n = 681 653	**Adjusted model 2**<br>n = 681 653
	RR [95% CI]	RR [95% CI]	RR [95% CI]
SER	Ref	Ref	Ref
Substandard	1.76 [1.67 – 1.86]	1.29 [1.22 – 1.37]	1.29 [1.22 - 1.37]
PE	2.38 [2.15 – 2.62]	1.57 [1.42 – 1.74]	1.58 [1.42 – 1.75]
		**Age group 57 – 65**	
	**Crude**<br>n = 557 183	**Adjusted model 1**<br>n = 556 273	**Adjusted model 2**<br>n = 556 273
	RR [95% CI]	RR [95% CI]	RR [95% CI]
SER	Ref	Ref	Ref
substandard	1.53 [1.47 – 1.60]	1.15 [1.09 – 1.20]	1.14 [1.10 – 1.20]
PE	1.85 [1.72 – 1.98]	1.32 [1.23 – 1.42]	1.31 [1.24 – 1.42]

Note: Model 1 adjusted by age, sex, level of education, days in unemployment during the last year, family income as percentage of the median income Model 2 adjusted by age, sex, level of education,

days in unemployment during the last year, family income as percentage of the median income and exposure to chemicals and fumes (JEM).
SER: Standard Employment Relationship (score: 0 or less than 0) Substandard (score between -1 and -3) and PE: Precarious employment (score below -3).

Table S5. Associations between exposure to levels of PE (2003) and risk of COPD (2004-2017) among individuals with complete data on smoking, separated by age groups (40 – 48, 49 – 56, 57 – 65).

Women (N = 391.744) Model 3			
	**Age group**<br>**40- 48**<br>**N = 267 825**	**Age group**<br>**49 – 56**<br>**N = 111.469**	**Age group**<br>**57 – 65**<br>**N = 12.196**
	RR [95% CI]	RR [95% CI]	RR [95% CI]
SER	Ref.	Ref.	Ref.
Substandard	1.17 [1.00 – 1.37]	1.34 [1.14 – 1.59]	1.42 [0.98 – 2.05]
PE	1.34 [1.03 – 1.75]	1.95 [1.49 – 2.54]	2.13 [1.25 – 3.61]
Missing: 254			
Men (N = 33.277) Model 3			
	**Age group**<br>**40- 48**	**Age group**<br>**49 – 56**<br>**N = 33.172**	**Age group**<br>**57 – 65**
	RR [95% CI]	RR [95% CI]	RR [95% CI]
SER		Ref.	
Substandard	-	1.24 [0.93 – 1.65]	-
PE	-	1.48 [0.86 – 2.53]	-
Missing: 105			

Note: Model 3 adjusted by age, sex, level of education, days in unemployment during the last year, family income as percentage of the median income exposure to chemicals and fumes (JEM) and smoking.
SER: Standard Employment Relationship (score: 0 or less than 0) Substandard (score between -1 and -3) and PE: Precarious employment (score below -3).
Complete smoking data is available for men from the Swedish Conscription register for the year of (1969), and the smoking data for women is available from the Swedish Medical Birt register (SMBR) and for the years 1983 -2003.

Table S6. Associations between exposure to levels of PE (2003) and risk of COPD (2004-2017) among individuals without occupational exposure to chemicals and fumes.

Men		
	**Crude**<br>(N=829.346)<br>RR [95% CI]	**Adjusted model 1**<br>(N=827.419)<br>RR [95% CI]
SER<br>n= 21 907	Ref	Ref
Substandard<br>n= 9 972	1.83 [1.73 - 1.93]	1.34 [1.26 – 1.41]
PE<br>n= 1 284	2.67 [2.45 - 2.91]	1.50 [1.37 – 1.64]
Women		
	**Crude**<br>**(**N= 990.041)<br>RR [95% CI]	**Adjusted model 1**<br>(N = 988.920)<br>RR [95% CI]
SER<br>n=166 497	Ref	ref
Substandard<br>n= 202 532	1.53 [1.47 - 1.60]	1.08 [1.03 - 1.13]
PE<br>n= 10 715	2.01 [1.87 - 2.16]	1.26 [1.17 - 1.36]

Note: Model 1 adjusted by age, sex, level of education, days in unemployment during the last year and family income as percentage of the median income.
SER: Standard Employment Relationship (score: 0 or less than 0) Substandard (score between -1 and -3) and PE: Precarious employment (score below -3).
Information on occupational exposure is based on the SWE-JEMS, based on probability of exposure on an aggregated level, with a cut-of o 50%.

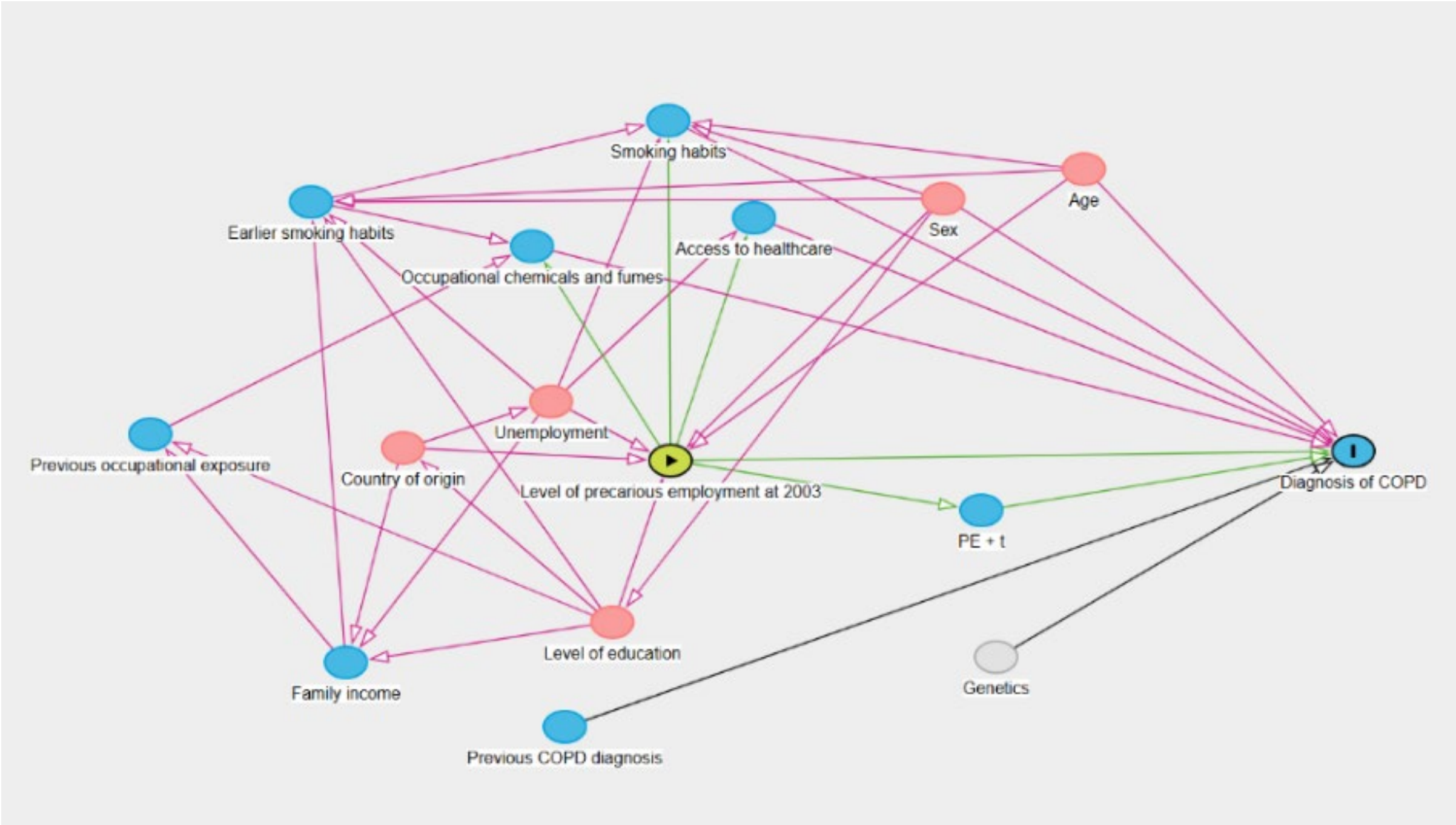

Figure S1. Directed acyclic graph (DAG) of the relationship between precarious employment and COPD.

Note: DAG illustrating the assumed relationship between PE in 2003 and subsequent diagnosis of COPD. Arrows indicate hypothesized casual pathways. Potential confounders included age, sex, country of origin, education, family income, unemployment, smoking habits, occupational exposures, and access to healthcare.
